# Dr. Allen’s Improvement

**Published:** 1852-04

**Authors:** 


### Editorial.

### DR. ALLEN'S IMPROVEMENT.

We are frequently written to in relation to Dr. Allen's method of fastening teeth to plates. We have said but little heretofore on this subject, wishing to more fully test the strength of adhesion to the plate and its availability in general practice.

It is now some six months since we had operations put up in this way, and we will give the result of our experience and observation, judging that this will be more acceptable to the profession than any theorizing we might be able to give.

The plates we have used are platinum. This is a little heavier than gold; we judge it will add in weight to an upper plate some two dwt. It swedges more readily to the cast than gold, and we think in everything is equal to gold for dental purposes but in weight and color.

One of the first operations we had put up in this way was under the following circumstances. We had inserted on gold plate an atmospheric upper set. The teeth required for this operation were the smallest size of gum teeth we could procure, and the gum had to be ground so thin that we were fearful it would break away. In soldering them to the plate it was slightly warped. This was enough to injure some their utility, and instead of remodelling this to suit our patient, we determined to test the continuous gum of Dr. Allen's on platinum plate. After this was swedged to fit the mouth, we selected a set of very small teeth, adjusted them to the plate with about ten minutes' grinding, and having secured them to the position we desired with wax, tried them in the mouth and saw that every tooth was in its right place for a perfect articulation. This done, it was imbedded in plaster, sand and asbestos, or a compound which the Dr. uses for that purpose, and as the teeth were small and the entire bite on the first ten teeth, we attached small platina backs to the four bicuspids. The operation was then prepared with cement and gum and put into the furnace for baking. When taken out in appearance it equalled our utmost expectations, and when tried in the mouth no appreciable change of fit was perceptible. This operation we have carefully watched ever since, and find as yet no flaw or crack about it, and although they are used for mastication at every meal, yet not the slightest change is apparent in the adhesion of the gum. We have had several operations which have proved as satisfactory, but which required a larger amount of cement and gum to be flowed in around the teeth. We have in some of these used lining straps to the teeth, in others not. When they are used we line up the teeth with our scraps of platina plate, and solder up with pure gold; then flow the cement and gum on the labial surface, and between and under the teeth. This makes a very neat operation, and with the present style of teeth is as good a plan as any other. We hope however, that we shall have teeth made with special reference to this method of fastening to plates, and the palatal face of the tooth made to resemble the natural organ.

The case we have described we selected as a test operation, and determined, if it answered, to pursue the practice wherever our patients had no preference for the old mode.

We have had far less of warping of plates than before, and indeed in no case has this been such as to render it necessary to take off the teeth and swedge up again. We have now given the result of those operations, which have given us confidence in the plan as practised by Dr. Allen, and we will give also the result of the most unfavorable case. Some six or eight months since we put up a set for a gentleman, upper teeth on atmospheric pressure principle: some of the teeth had very light straps put on and the cement flowed over them: and when finished and put in the mouth, promised to do well, but in two or three weeks the gentleman had broken off three or four of the teeth and several pieces of the gum; some of the teeth broke off near the plate, taking portions of the gum with them, others had broken at their necks. We broke up the operation, and found the cement and gum in places perfectly attached to the plate, and in other places imperfectly. We then again swedged up the plate, selected other teeth, and fastened them as before. Some four months have now passed, and one tooth is broken off, the lateral incisor, near or at the upper pin, and in one place the gum has cracked and a scale flared off. This we understand was occasioned by the teeth falling from the top of a bureau to the floor or hearth. This operation is temporary, having been put in a few days after the extraction of the teeth, and the gums are so much changed as to very much lessen the suction. In the first instance we think the teeth had been injured by the heat, and that the plate was not properly prepared. The like accident might however happen to any set of teeth, and often does. The inquiry would be however, how can it be remedied?

This is very easily done: on the corundrum wheel grind off the broken tooth and gum nearly to the plate, select another tooth, apply more gum material, and heat up again, and if you use the same colored gum no one could perceive the place where the break had been. In one case the six upper front teeth proved when inserted, too short. We broke them off with a hammer, and then ground off nearly all the cement and gum, selected longer teeth, and flowed in cement and gum around these, uniting the whole, and making the operation as perfect as ever. In all cases where the plate has to be united by soldering, we use pure gold, and we have not found that the flowing of the enamel injures the union effected by the gold as a solder.

We generally turn up the outer border of the plate for a rim, which gives a wired edge to the operation, and prevents cutting of the integuments. We have not as yet used the improvement in partial sets, (except for partial under-sets) yet we think where much gum is necessary it would be very useful and convenient, or with it unite several teeth into a block, and solder them to a gold plate.

That the gum may occasionally crack from the plate and small pieces scale off, we do not doubt. Absorption of the gum, which should very much change the basis on which the plate is to rest, would necessarily cause a heavy strain on the piece at some point or other. This sometimes will occasion a break in a very strong gold plate, but we doubt if the gum will scale from the teeth and plate as much as the gum from the ordinary gum teeth ; in either case that which is very thin must be more liable to break than the thick.

We have no doubt but that some considerable manipulation is necessary to put up neat operatious in this way, yet when done in the most perfect manner, they certainly present a neatness and finish which is not arrived at in the old method. In conclusion, we would say, that we have been watching the development of this process with considerable interest and some doubt as to its permanent success. These doubts have been gradually giving way until we feel no longer any hesitancy in adopting it in our practice.

